# Recruitment and Retention of Urban Pregnant Women to a Clinical Study Administering an Oral Isotope Dietary Tracer

**DOI:** 10.1089/whr.2022.0015

**Published:** 2022-07-18

**Authors:** Mary Dawn Koenig, Lisa Tussing-Humphreys, Victoria DeMartelly, Bazil LaBomascus, Nefertiti OjiNjideka Hemphill, Lauren Welke, Lacey Pezley, Rungnapa Ruchob, Bruni Hirsch, Melissa Furlette-Koski, Nicollette Kessee, Carol Estwing Ferrans

**Affiliations:** ^1^ Department of Human Development Nursing Science, College of Nursing, University of Illinois Chicago, Chicago, Illinois, USA.; ^2^ Department of Kinesiology and Nutrition, College of Applied Health Sciences, University of Illinois Chicago, Chicago, Illinois, USA.; ^3^ Department of Medicine, Northwestern University, Chicago, Illinois, USA.; ^4^ Department of Medical Science, Abbvie, Chicago, Illinois, USA.; ^5^ Department of Obstetric and Gynecological Nursing, Mahidol University, Bangkok, Thailand.; ^6^ Department of Midwifery, Saint Anthony Hospital, Chicago, Illinois, USA.; ^7^ Department of Midwifery, St. Joseph Mercy Health System, Ann Arbor, Michigan, USA.

**Keywords:** iron isotopes, maternal–fetal exchange, obesity, patient selection, pregnancy, recruitment

## Abstract

**Introduction:**

Pregnant women are a vulnerable population that are difficult to engage in clinical research. We report successful recruitment and retention strategies used in a longitudinal pilot study of urban racially/ethnically diverse pregnant women that involved administration of an orally ingested isotope tracer, multiple venipunctures, biopsy of placenta after delivery, and cord or placental blood collection.

**Materials and Methods:**

We used direct strategies to recruit English-speaking obese and nonobese pregnant women aged 17–45 years, who were in the third trimester of pregnancy. The study required data collection at 32–34 and 34–36 gestational weeks and delivery. Strategies included frequent personal engagement with participants and staff to build relationships and trust, tangible appreciation, and the study team being present at delivery. In addition, leveraging hospital information technology (IT) services was critical to ensure retention through labor and delivery (LD).

**Results:**

A racially (52% Black, 23% White, and 10% other) and ethnically (15% Hispanic or Latinx) diverse sample of pregnant women was enrolled. Of the 52 women enrolled, 85% of women completed all procedures.

**Conclusions:**

This is the first report of successful strategies for recruitment and retention of racially/ethnically diverse pregnant women in a longitudinal study requiring oral administration of an isotope tracer. Personal engagement with multiple touch points, starting with recruitment and continuing regularly throughout the third trimester, was the most successful strategy. Creating and maintaining relationships with the LD providers and staff and utilizing hospital IT, including targeted electronic medical record alerts, ensured successful retention for the duration of the study.

**Trial Registration:**

Not applicable.

## Introduction

National Institutes of Health (NIH) and the NIH Office of Research on Women's Health have called for research to promote evidence-based clinical practice related to pregnancy.^1^ However, pregnant women are a vulnerable population that are particularly difficult to enroll in clinical research because of the perceived potential harm to the fetus, including unforeseen teratogenic effects and adverse pregnancy outcomes.^2^ Underserved populations are often at higher risk in pregnancy, but historical mistreatment by the medical and scientific community has decreased willingness to participate.^3–5^ Urban low-income women may face additional challenges such as safe and accessible transportation, inflexible work schedules, and affordable childcare.^6^

Other disincentives add to the difficulty of recruiting pregnant women. Given the length of gestation, participation for a prolonged period may be required, especially if the research includes infant health outcomes.^7^ Burdensome protocols with multiple face-to-face interactions and/or complicated procedures may also dampen enthusiasm.^8^ The use of procedures not typical of routine clinical care may frighten potential participants. This may be especially true of studies that collect samples during labor and delivery (LD), including the placenta and cord blood, which may be perceived as an invasion of privacy. Moreover, pregnant women are often overwhelmed with other household, childcare, and work responsibilities, complicating participation.^6^

We report successful recruitment and retention strategies used in a longitudinal pilot study among urban racially/ethnically diverse pregnant women, requiring administration of an oral dietary isotope tracer, multiple venipunctures, biopsy of placenta after delivery, and cord or placental blood collection. Previous studies have reported strategies for engaging urban pregnant women in either a behavioral intervention^9–12^ or epidemiological studies with no intervention^13–15^ with recruitment rates ranging from 54% to 85% among Black^9,12,13^ and 19% to 52% among Hispanic^15,16^ populations, and retention rates of 53% for Black^12^ and 65% for Hispanic^16^ populations.

Studies included a smoking reduction intervention scheduled to coincide with prenatal care visits for four sessions lasting an average of 35 minutes^12^ and prevention of dental caries intervention including an oral examination, surveys, and counseling.^16^ Other studies among predominantly White pregnant people report recruitment rates of 20% for survey completion,^14^ 28% for two electronic interventions for weigh management,^11^ 33% for four pelvic examinations,^17^ and 55% for a venipuncture, cord blood collection, and a 4-hour time commitment.^7^ Studies among predominately White pregnant people report retention rates of 43%^17^ and 89%.^7^ However, to our knowledge, none have reported strategies for engaging and retaining urban pregnant women using more invasive research methods such as a dietary isotope tracer and collection of blood and tissue at delivery.

## Methods

### Study design

**FIG. 1.**
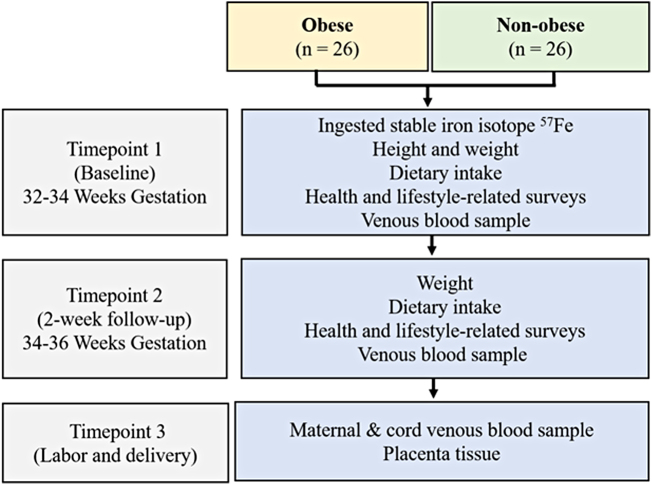
Study design.

This was a longitudinal study of obese and nonobese pregnant women to determine how maternal prepregnancy obesity affects maternal iron absorption and placental iron transfer using a stable iron isotope (*i.e.*, dietary tracer). Details regarding the research visits, study-related procedures, and data obtained at each visit are described in Figure 1.^18,19^ The University of Illinois at Chicago (UIC) Institutional Review Board approved the study procedures (No. 2015-0353). Our goal was to enroll 52 pregnant women (26 obese and 26 nonobese). The study involved three research visits.

At baseline (32–34 weeks gestation), women provided a blood sample, height and weight were measured, health and lifestyle-related surveys were completed, and a nonheme iron solution labeled with the stable iron isotope ^57^Fe was administered orally.^18^ Two weeks later (34–36 weeks gestation), women returned for a second research visit at which maternal weight and dietary intake were assessed and a venous blood sample was collected. The final research encounter took place during LD. At this time, a cord blood sample was collected to examine placental transfer of the maternally ingested stable iron isotope, as well as the placenta to explore potential differences in the expression of genes and proteins related to placental iron uptake and transfer between the obese and lean women.^19^

#### Inclusion and exclusion criteria

Eligibility for the study included singleton pregnancy, 17–45 years old; prepregnancy body mass index ≥18.5 kg/m^2^. Women were excluded for any health conditions or medications that may impact study outcomes (details in publication of maternal outcomes).^18^

### Recruitment strategy

#### Initiating relationships with clinical staff at the Center for Women's Health

Before commencing recruitment, the study team met in-person with the clinic medical manager, clinic medical staff, and all the members of the midwifery practice to introduce the study, determine the optimal methods for recruitment, and avoid disrupting care and patient flow. Recruitment procedures were discussed, actively eliciting recommendations from the clinical staff and midwives regarding their preference for recruitment (meet patient in waiting room vs. examination room) and suggestions for enhancing patient engagement.

#### Unique study logo

A memorable logo developed for this study provided a strong nonverbal cue (for recognition of the study and as a reminder) for staff and participants. Throughout the study, the logo was included on all printed materials: recruitment flyers, flyers for staff, instructions for participants, and posted signage throughout the LD unit. We also included headshots of the study team on materials whenever possible.

#### Information technology access and training

All research team members received access to the University of Illinois Hospital electronic health records and clinical schedules. Research team members used protocols developed for the study to screen the clinic schedules and patient medical records for eligible patients.

### Recruitment activities

Clinic schedules and patient medical records were reviewed using a prescreen eligibility checklist. Women deemed eligible were approached during a late second or early third trimester appointment in the waiting area or a patient examination room. Interested and eligible women were asked to provide contact information so that the study primary investigator (PI) could follow up personally.

The PI contacted each woman by phone to confirm interest and provide a more detailed description of study procedures. Once a participant agreed to participate, the PI obtained her due date and determined possible baseline study visit dates between 32 and 34 weeks gestation. Often participants would schedule the baseline research visit to coincide with a scheduled clinic visit. The research center was conveniently located in a building minutes from the clinic, accessible through an indoor pedway. Once the baseline research visit was scheduled, the PI contacted the participant *via* text and e-mail a week in advance to remind them of the upcoming study visit. The reminder e-mail contained directions (both driving and public transport), a detailed map, and instruction regarding how to prepare for the visit. Participants were asked to fast for 2 hours before the visit, refrain from eating iron-rich foods before the visit, and consume a standard meal after the visit.

### Other relevant recruitment-related activities

#### Guidance for culturally sensitive recruitment

The Community Engagement Advisory Board of the UIC Center for Clinical and Translational Sciences (Grant No. UL1TR002003) met with us to provide guidance for presenting the study to potential participants, as well as retaining them, and reviewed the consent form and study flyer. Key feedback included (1) refraining from using clinical research language, including using lay language for the isotope, (2) presenting the study from the perspective of the knowledge to be gained, and (3) potential advances in care that would help other women in the future.

#### Regular in-service for clinical staff

Our team met regularly with the clinic staff to provide updates on progress and to receive feedback for better integrating our study into the clinic workflow. Whenever possible, all team members, including the PI, research assistants, and students, attended to increase engagement. Tokens of appreciation, such as handwritten cards expressing gratitude and home-baked goods, were given to staff at meetings and intermittently throughout the study.

### Retention strategies

Because women engaged in the study from 32 to 34 weeks gestation through delivery, retention was extremely important. We employed a variety of retention methods targeted at both research participants and LD clinical staff (Table 1).

**Table 1.** Key Strategies for Recruitment and Retention

	Engagement with participants	Engagement with health care providers (clinic and hospital)
Formative feedback	Community Engagement Advisory Board provided culturally sensitive guidance for recruitment strategies	Guidance elicited from clinic and LD staff regarding recruitment and conduct of study
Personal engagement (building relationships and trust)	Multiple touch points with participants (in person, e-mail, text, and phone), throughout third trimester, starting with recruitment	Regular in-person meetings with clinic staff and LD staff and providers (initial study in-service and periodic updates)
Printed materials (all branded with unique study logo)	Recruitment flyers Maps and instructions Delivery instruction card	Study updates for staff, including photos of staff Posters posted throughout LD unit, including patient rooms
Tangible appreciation	Modest participant reimbursement	Home baked sweets brought to clinic and LD staff meetings Personal thank you notes with photos of study staff delivered personally to clinic and LD staff
Information technology		Access to the EHR and patient records by study staff: • Identify potential participants • Evaluate clinical changes • Identify upcoming appointments to coordinate with clinical care • Monitor obstetrical emergency room triage and LD admissions Automatic alerts: • To LD unit staff, notifying of patient's participation in study • To study team that participant was admitted to LD unit
Engagement at delivery	Delivery packet containing: • Laminated instruction card for patient, with phone numbers to call staff and for ride to hospital • Vacuum tubes for blood draw • Laminated instruction card to be given to staff Study staff member present at delivery	Study staff member present at delivery Called LD unit intermittently to receive updates on participant status and create a point of contact with LD nurse. Addressed providers by their name

EHR, electronic health record; LD, labor and delivery.

#### Retention methods for study participants

At the baseline research visit, we confirmed contact information for the participant, and collected contact information of another family member or close friend. We also confirmed that the patient had no plans to move out of the Chicagoland area and intended to deliver their baby at the University of Illinois Hospital. The study PI and/or a member of the research team was present at the baseline visit, to develop a personal connection between the study team and research participant.

The week before the first follow-up visit, our team sent an e-mail reminder detailing the date, time, and location (including a map) of the follow-up appointment and study-related instructions. The day before the study visit, subjects were called by phone, e-mailed, and/or sent a text message with a reminder. If visit confirmation was not received, a member of the research team met the participant at their clinical appointment to remind them of the study visit after their appointment. For all participants, the study PI and/or a member of the research team was present at the follow-up visit, again bolstering the connection between the subject and research team.

During the subsequent 2-week follow-up visit, a research team member again confirmed the subject's contact information and that the patient intended to give birth at University of Illinois Hospital. At the end of the visit, each woman was provided a laminated card and blood collection tubes packaged in a biohazard bag to be used when they were admitted to LD. Women were asked to put the laminated card and blood collection tubes in their purse or packed hospital bag. The laminated card would inform the LD clinical staff that the participant was in a research study, including a study number so that clinical staff could contact the researcher on call regarding the admission; instruction to draw blood into the provided tubes upon admission was also included. We also placed signage throughout the LD unit with study instructions for nurses, as well as on our study supplies bin in the LD laboratory room.

In addition, a medical transportation number was provided on the laminated card. This number was a free service provided by the hospital to women in labor. If an ambulance was called by the participant instead, they would be transported to the nearest local hospital, and we only had privileges to collect research samples at delivery at the University of Illinois Hospital.

After the 2-week follow-up visit, communication was maintained until admission for delivery through phone calls, e-mails, and text messages. There was an average of four touch points conducted personally by the PI between the follow-up visit and admission for delivery. During each communication, the participant was reminded to call or text the study number if they were going into the hospital, as well as genuine enthusiasm for their upcoming delivery and arrival of the baby. Text conversations occurred every 2 weeks after the follow-up visit and then weekly after the participant reached 38 weeks gestation.

#### Financial incentives

Financial incentives also were provided throughout the study in cash ($75 at baseline, $25 at 2-week follow-up visit, and $50 at LD visit) totaling $150. Other studies report providing $15 in cash for collection of a genital tract specimen and completion of surveys with recruitment rates of 57% in a mixed race sample.^13^ Others report incentives up to $140 in cash with eligibility to earn an additional $150 for completion of all study-related activities with a recruitment rate of 28% and retention rate of 85% during pregnancy and 70% after delivery in a predominantly White sample (63%).^11^ Zielinski et al report providing a total of $260 in cash across four visits ($40 at the first, $40 at the sedon, $80 at the third, and $100 at the fourth) with a 33% recruitment rate and 43% completion rate in a predominantly White (87%) pregnant population.^17^

After each of the nine visits, participants in a prevention of dental caries trial received $20 grocery voucher ($180 total), free dental cleaning, and discounted dental services with a 19% recruitment rate and 65% completion rate in predominately Hispanic (92%) population.^16^ Finally, in a population of Black pregnant people participating in a randomized controlled trial of smoking reduction across four sessions lasting 35 minutes, participants were reimbursed a total of $100 ($5 to complete the screening survey, received a 30-minute telephone card, $15 for an interview, $10 for each intervention session, and $15 and $25 for two postpartum sessions) with recruitment rates of 85% and retention of 54%.^12^

#### LD clinical staff training to enhance retention

The study investigators met with the obstetrical care providers, including the attending physicians, resident physicians, nurse-midwives, nurses, and unit staff before initiating the study. Subsequently, periodic in-services were conducted, with all study team members present. The time-sensitive nature of the cord blood and placenta collection was discussed and the need for the researchers to be present and in the room during the birth.

Assistance from the provider to collect anything beyond the maternal blood sample upon admission was not requested, given that it could impact the timing and flow of clinical care. However, the study team simply asked that the providers make the placenta available and keep the cord clamped for research sample collection. In addition, the study phone number was placed on a placard located next to the supplies in each LD room and in each obstetrical triage room, instructing the staff to ask if their patient was in the research study. This placard was branded with the unique study logo.

#### Information technology solutions to enhance retention

The study investigators worked with a member of the University of Illinois Hospital Information Technology (IT) team to create an automated message to be triggered in the medical record system when the participant was admitted to the LD unit. Specifically, a message was printed for the LD nursing staff indicating the participant was enrolled in the study and to call the study number and alert the study team. The IT team also programmed an automated message that alerted the study team when a participant was admitted. By leveraging hospital IT, the study team was able to monitor prenatal appointments between the follow-up visit and delivery, which helped to identify inductions, and scheduled cesarean sections. The research team was also able to closely monitor the obstetrical emergency room triage and LD unit admissions.

#### Additional methods to enhance retention

Two research team members were on call 24 hours/day, 7 days per week. During an on-call shift, the emergency room obstetrical triage and LD unit admission list was checked every 2 hours, through the hospital online portal. If a participant was in triage or admitted, a team member would go immediately to the hospital.

## Results

We screened 3473 women between August 2015 and August 2017. Table 2 indicates reasons for study ineligibility. The top reason for ineligibly was gestational age (*n* = 626) and body mass index out of range (*n* = 579).

**Table 2.** Reasons for Exclusion

Exclusion criteria	Screened
Gestational age out of range	626
Body mass index out of range	579
Race	446
Pregnancy loss/abortion/no documentation of pregnancy	214
Pregnancy in past 12 months	154
Language other than English	141
Approached and declined	114
Parity ≥3	112
No show/missed/changed care	111
Interested but never enrolled	91
Tobacco use in the past 3 months/current alcohol or drug use	83
Patient tracked/lost to follow-up	77
Asthma/steroid use	76
Previous preterm birth/current or previous premature rupture of membranes	76
Hematologic disorder	71
Current bacterial/viral infection/antibiotic use	62
Age out of range	52
Enrolled	52
High-risk pregnancy (*i.e.*, preeclampsia, hypertension, placenta previa, hypothyroidism, polycystic ovarian syndrome, autoimmune disorder, ectopic pregnancy, prolactinoma, and retained IUD)	45
Fertility treatment	40
Pregnant with multiples	31
Not seeing a midwife for prenatal care/not delivering at UIC/already delivered	26
Current/previous gestational diabetes or previously diagnosed type 1 or 2 diabetes	17
Inadequate weight gain	16
Bariatric surgery/GI disease	5
Placenta encapsulation	1
No reason recorded	155
Total	3473

GI, gastrointestinal; IUD, intrauterine device; UIC, University of Illinois at Chicago.

From August 2015 to August 2016 we focused our recruitment efforts on Black women and excluded other races, resulting in 446 women being excluded. Beginning August 2016, we began recruiting all races/ethnicities. Other common reasons for exclusion included a pregnancy loss, abortion, no documentation of pregnancy (*n* = 214), another pregnancy in the past 12 months (*n* = 154), unable to speak English (*n* = 141), and parity of three or more (*n* = 112).

Of the 368 women who prescreened eligible, 257 were approached in the clinic and 114 declined to participate, resulting in a 20% enrollment rate (Table 3). Within each racial and ethnic category, 20% of Black, 30% of White, 17% other, and 21% Hispanic/Latinx individuals who were eligible and available (did not miss an appointment and were approached by the study team) enrolled in the study.

**Table 3.** Racial/Ethnic Background of Potential Participants

Race/ethnicity	Screened	Eligible	No show/missed/changed care	Interested but never enrolled	Declined	Enrolled
Black	1492	182	48	44	63	27
Unavailable	639	23	15	4	4	0
White	406	65	25	12	16	12
Other	392	43	13	11	14	5
Hispanic/Latinx	366	48	10	18	12	8
Asian	160	6	0	1	5	0
Native Hawaiian	9	1	0	1	0	0
American Indian	8	0	0	0	0	0
Pacific Islander	1	0	0	0	0	0
Total	3473	368	111	91	114	52

Women who prescreened eligible but did not attend their scheduled visit or switched care providers were not approached (*n* = 111). We received contact information from 143 women, and all 143 were called, and a text message was sent to ascertain interest within 1 week of being approached in clinic. Of those 143 women who were called, 65 scheduled a baseline appointment and 52 attended the baseline visit and were enrolled in the study. We enrolled a racially (52% Black, 23% White, and 10% other) and ethnically (15% Hispanic/Latinx) diverse sample of women, which is reflective of the population that the clinic serves.

Regarding the 52 women enrolled, 1 was lost to follow-up between baseline and the follow-up visit and 7 were lost between the follow-up visit and the LD visit, with 44 women completing the study (85%). Among the women lost to follow-up between the follow-up visit and LD (*n* = 7), four women delivered at a hospital other than the University of Illinois Hospital.

## Discussion

To our knowledge, ours is the first report of recruitment and retention strategies used in a longitudinal study requiring oral administration of a stable isotope dietary tracer in pregnant women. Fifty-two of the 257 eligible women approached in the clinic enrolled (20%) and is comparably low to enrollment rates of pregnant women in other studies (19%–85%).^9,10,12,16^ However, some of these studies required only a behavioral intervention or were observational, unlike ours that required a potentially frightening and ingested isotope, multiple venipunctures, biopsy of placenta after delivery, and cord or placental blood collection. In fact, early reviewers of our study expressed concern that we would be unable to find any pregnant women willing to participate.

Nevertheless, once a participant enrolled in our study, we were able to retain them through to delivery (85%). This retention rate is comparable with a study evaluating an electronic intervention for weight management with an 85% retention throughout pregnancy.^11^ Other studies in pregnancy report retention rates ranging from 43% to 89%.^7,12,16,17^ In fact, three of the four women who delivered at other hospitals outside UIC alerted us to their arrival at those hospitals and requested our presence at the delivery. This speaks to the commitment that our participants had to completing the study once enrolled.

Documented historical mistreatment of underserved populations by the medical and scientific community, particularly Black individuals, has increased distrust and decreased willingness to participate in research.^3,20^ Given the fact that our study required ingesting a dietary tracer and additional procedures at delivery, it would be expected that communities of color would be reluctant to take part. However, we found that communities of color were represented and enrolled in the study. In fact out of those individuals who were eligible and approached by the research team, 20% of Black, 30% of White, 17% other, and 21% Hispanic/Latinx individuals enrolled in the study. Communities of color can be successfully recruited and retained in a complex and potentially frightening study such as ours.

The close relationships with providers and medical staff that we cultivated throughout the study were essential to the success of recruitment and retention.^7,21^ Regular in-services with progress updates, both with the clinic and LD staff, kept them engaged. Building and maintaining these relationships was time-intensive, but a high-yielding strategy with 100% of the enrollment through this direct recruitment method. Direct recruitment is an essential recruitment strategy for pregnant women,^22^ especially among low-income women of color, and allows the research staff to answer any questions immediately to quell fears the participants or their families may have regarding procedures. This is especially important when a study is requesting sensitive tissues and samples, such as placenta and cord blood.

Our team found that moderate financial incentives and multiple interactions, including text messages and in-person meetings, with consistent research personnel, developed rapport between the research staff and participants. In fact, often significant others or mothers of the participant would contact the research team to update us on their arrival to the hospital. This meaningful relationship between research staff and participant promoted trust and instilled confidence in the research team.^23,24^ At time of consent, discussing how the results would be used in future studies and potentially inform practice change allowed the participants to make connections with “real-life” clinical applications.

### Limitations

In an effort to isolate obesity's impact on maternal iron absorption and placental transfer, we created strict inclusion and exclusion criteria, which severely limited the number of eligible women. Of the women that we prescreened, only 11% were deemed eligible. For example, women with health conditions that may impact study outcomes were excluded. In addition to small sample size, participants were all recruited from one urban hospital, all of which may limit generalizability.

## Conclusions

Collection of placenta and cord blood samples is inherently challenging due to the emergent nature of delivery. We were able to recruit and retain a racially and ethnically diverse sample of pregnant women for a longitudinal study using a stable isotope, multiple maternal venipunctures, and collection of cord blood at delivery in addition to biopsy of placenta tissue. We worked closely with the clinic and LD staff and practitioners while creating a meaningful and trusting relationship with our participants to ensure retention to delivery. Access to medical records and unit census was critical to detecting when participants arrived in the LD unit. The use of multiple modes of communication among staff, practitioners, and participants and monitoring of the medical record ensured success of the study.

Recruitment and retention of pregnant women in research is a challenge, particularly in low-income urban populations. Although the work is time-intensive, the barriers to inclusion are not insurmountable. With almost 4 million births in the United States each year, it is essential to include representative populations in studies, so that findings will inform clinical practice and public policy, particularly for those most at risk.

## Abbreviations Used

EHR: electronic health record
IT: information technology
LD: labor and delivery
NIH: National Institutes of Health
PI: primary investigator
UIC: University of Illinois at Chicago
